# The flow Rate Accuracy of Elastomeric Infusion Pumps After Repeated Filling

**DOI:** 10.5812/aapm.14989

**Published:** 2014-04-07

**Authors:** Masood Mohseni, Amin Ebneshahidi

**Affiliations:** 1 Department of Anesthesiology, Rasoul Akram Medical Center, Iran University of Medical Sciences, Tehran, Iran; 2 Persia Research Center, Isfahan, Iran

**Keywords:** Elastomers, Infusion Pumps, Reliability, Dimensional Measurement Accuracy, pain

## Abstract

**Background:**

One of the frequent applications of elastomeric infusion pumps is postoperative pain management. In daily practice, the disposable pumps get refilled with modified medication combinations in the successive days; although, the accuracy of infusion rates is unknown to clinicians.

**Objectives:**

Our aim was to evaluate the effect of repeated filling on the delivery rate accuracy of an elastomeric pump available in our market.

**Materials and Methods:**

We examined 10 elastomeric infusion pumps (BOT-802, Nanchang Biotek Medical Device Company, China) with 100 mL capacity and nominal flow of 5 mL/h. Each pump was filled for three times, accounting for 30 series of experiments. A microset scaled in mL was used to measure the pump deliveries. Flow profile and reliability of infusion rate were analyzed after repeated use.

**Results:**

The mean flow rate in the three series of measurements showed a gradual increase; however, the difference was not statistically significant (5.01 ± 0.07 vs. 5.03 ± 0.06 vs. 5.06 ± 0.08 mL/h; P = 0.81). The percentage of the flow rate error (deviation from 5 mL/h ± 15%) was 100% in the first and second hours of infusion, 96% in the third hour, 60% in the 20th hour and zero percent in the rest of the infusion time.

**Conclusions:**

This study indicated that the delivery rate accuracy of elastomeric infusion pumps is preserved after repeated usage. These laboratory findings suggested that elastomeric pumps could be safely refilled in the successive days to provide postoperative analgesia.

## 1. Background

Elastomeric infusion pumps have gained wide acceptance to provide postoperative analgesia in the recent years (1). Elastomeric infusion pumps provide superior postoperative analgesia than bolus dosages of opioids with less complications (2). They also exert several advantages over electronic pumps including portability, feasible demonstration, and fewer human errors with the setup of these devices (3, 4). Recent studies suggested that patients prefer elastomeric devices rather than electronic pumps because of their low weight and size, ease of use, and less interference with sleep (5, 6). However, the use of elastomeric pumps is not fault free in spite of their several advantages. The delivery rate accuracy of these devices is low in comparison with modern electronic infusion pumps (7, 8). Nevertheless, detection of abnormal drug delivery is difficult due to the absence of alarms. These characteristics necessitate more consideration in the use of these devices.

Several elastomeric infusion pumps are now available from different manufacturers. They are calibrated in different conditions, including operating temperature and pressure, viscosity of fluid, backpressure, and time recommended between filling of the device and beginning of the infusion. All of these factors, mostly unknown to the end user, affect the infusion rate of pumps. The manufacturers reported flow rates within 15% of their set rates as acceptable (9-12). However, some earlier studies reported abnormal infusion times with resulting over sedation or inadequate analgesia (7, 8). Therefore, the delivery rate accuracy of different brands of elastomeric pumps should be tested in vitro before use.

The main criteria to select an elastomeric infusion pump for a certain clinical condition is the size of reservoir and its infusion rate. The flow profile of the pump should match the patient requirements and extend throughout the treatment. One of the frequent applications of elastomeric disposable infusion pumps is postoperative pain management. Noteworthy, in the early days postoperatively, there is a need for daily assessment of pain severity as well as complications of treatment, and subsequent daily adjustment of analgesic medications (13). Consequently, a large size pump for the whole period of analgesic treatment is not justified. On the other hand, replacing the administered elastomeric pumps in the consecutive days is economically unreasonable, especially in countries with limited financial resources. In daily practice, the pumps get refilled with modified medication combinations in the successive days; although, the reliability of infusion rates is unknown to clinicians.

## 2. Objectives

We conducted this study to evaluate the effect of repeated filling on the delivery rate accuracy of an elastomeric pump available in our market.

## 3. Materials and Methods

We examined 10 elastomeric infusion pumps (BOT-802, Nanchang Biotek Medical Device Company, China) available in our market. The capacity of the pump was 100 mL with the nominal flow rate of 5 mL/h. A microset with 100 mL capacity was used to measure the pump deliveries. One hole in the plastic cap of the microset was made, and the distal end of the catheter was inserted through this hole into the microset. Accordingly, the catheter tip of each pump was exposed to atmospheric pressure. The pumps were tested in 30-32 °C to simulate normal skin temperature. We concerned about possible fluid evaporation during the measurements. To test for potential evaporation loss, a microset with a hole in the plastic cap containing 100 mL of NS was placed in the same temperature and pressure conditions for 20 hours. At the end of examination, evaporation loss was less than 0.5 mL. We did not consider this volume in our analyses.

The manufacturer did not provide information regarding the calibration solution; therefore, we filled the pumps with normal saline (NS) 100 mL immediately before testing. The elastomeric pump and microset were placed in the same level. To start the infusion, perfusion tube clamp was released and the pump was primed according to the manufacturer’s instruction. We recorded the pump flow hourly using the collected volume of fluid in the microset until the complete deflation of pumps. The complete deflation was defined as at least 97 mL collected fluid in the microset. The subtracted three milliliters was the estimated residual volume in the administration tube set. After one hour of complete deflation, the pumps were refilled with normal saline and the tests were repeated. Each pump was filled for three times, accounting for 30 series of experiments. Global flow rate was computed as the measured volume in the microset divided by the time required for complete deflation of the pump. 

### 3.1. Statistical Analysis

Data are presented as mean and standard deviation or percentages, as appropriate. The global pump flow rate in three series of measurements was examined with Repeated Analysis of Variance. The percentage of the flow rate error was calculated as the measured flow rate minus five (nominal flow) divided by 5 and multiplied by 100. To evaluate the accuracy of infusion rate, percentage of flow rate error was calculated in assessment intervals (hours), and the 85-115% range was defined as acceptable. Standard deviation from the mean flow rate was considered as the indicator of the consistency of device performance. P < 0.05 was considered as statistically significant. Statistical analyses were performed by using SPSS version 16.0 software (SPSS, Inc., Chicago, IL, USA).

## 4. Results

### 4.1. Reliability of Pump Flow Rate After Repeated Use

The mean flow rate in the three series of measurements showed a gradual decline; however, the difference was not statistically significant (5.01 ± 0.07 vs. 5.03 ± 0.06 vs. 5.06 ± 0.08 mL/h; P = 0.81). Accordingly, the average finish time in the first, second and third trials were 19.46 ± 0.33, 19.39 ± 0.31 and 19.37 ± 0.31hours, respectively (P = 0.80).

### 4.2. Accuracy and Consistency of Flow Rate

**Figure 1.**
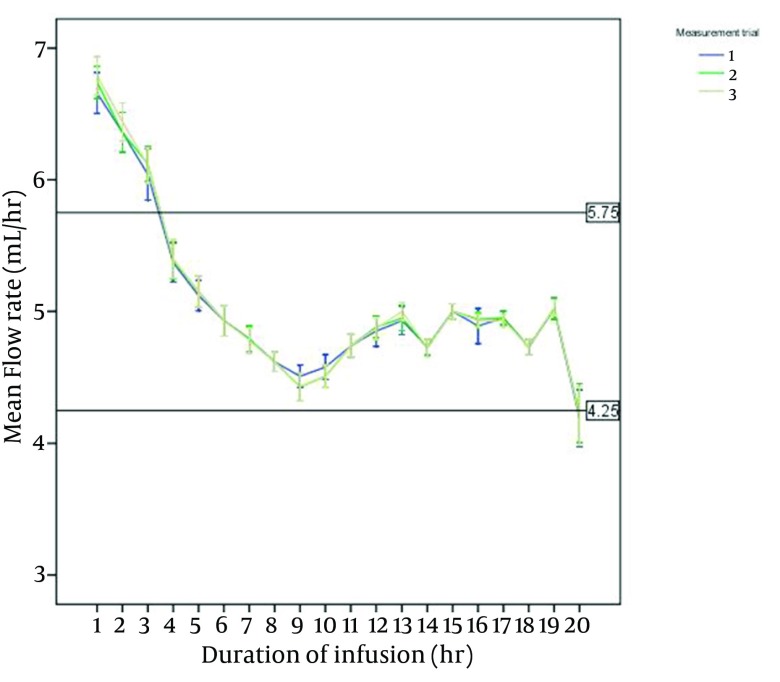
Delivery Flow Rates in Three Series of Measurement

Figure 1 shows the measured flow rates in one-hour intervals. The X-axis reference lines showed the acceptable flow rate (5 mL/h ± 15%). In all experiments, pumps initially infused at a rate faster than their nominal flow, and then returned closer to their set rates up to the complete deflation. The percentage of the flow rate error (deviation from 5 mL/h ± 15%) was 100% in the first and second hours of infusion, 96% in the third hour, 60% in the 20th hour and zero percent in the rest of the infusion time. Flow rate error in the initial hours of infusion was due to fast pump flows, and in the 20th hour due to slow infusion rates. Consistent pump performance as indicated by small standard deviations from the mean flow rate was preserved after repeated application. Pump flow occlusion, defined as flow rate less than 2.5 mL/h, was not observed in any of experiments.

## 5. Discussion

This study indicated that the delivery rate accuracy of elastomeric infusion pumps is preserved after repeated usage. The flow rate of the examined device (BOT-802) is accurate in most of the infusion time. These laboratory findings suggest that elastomeric pumps could be safely refilled in the successive days to provide postoperative analgesia. Elastomeric devices provide effective analgesia with fewer technical problems (14, 15) and expenses compared with electronic pumps (16, 17). This modification in the use of elastomeric devices makes their application economically more reasonable.

Elastomeric infusion pumps consist of a monolayer or multilayer elastomeric membrane, an outer protective shell, the administration tube set and a flow restrictor system. The stretched elastomeric membrane contains the fluid and generates the pressure required for delivery of medication. Manufacturers use various elastomers in their devices, both natural and synthetic (e.g. silicon, latex and isoprene rubber) (1). The type of elastomer, number of elastomeric layers and the geometry of the reservoir balloon determine the pressure generated on the fluid in a stretched balloon. The flow restrictor system mechanically limits the delivery rate to the set values. Elastomeric pumps generate a driving pressure of 260–520 mmHg and infuse at rates of 0.5-500 mL/h (1).

The manufacturers reported flow rates within 15% of their set rates as acceptable. However, earlier studies reported both high and low flow rates especially deflation defects by using elastomeric pumps (1, 8). Several factors may be involved in the inaccuracy of delivery flow rates. External pressure on the device (i.e. patient movements and positioning) accelerates the infusion rate in devices with soft shell. Conversely, it may induce occlusions of the tube set and failure to deliver the solution. Temperature also affects the infusion rate of elastomeric pumps. Most manufacturers calibrate the pumps at 31-32°C to simulate the normal skin temperature. Any variation in the temperature such as fever, warm suits or blankets would increase the infusion rate (4, 11, 18). In our in vitro study with controlled pressure and temperature conditions, the examined device (BOT-802) showed an accurate flow rate during most of the infusion time. The accuracy of delivery rate and consistency of performance were preserved after repeated filling.

The examined elastomeric pumps initially infused at a rate faster than their nominal flow, and then returned closer to their set rates up to the complete deflation. This flow pattern is common to all elastomeric pumps (18-20) and is due to variations in pressure within a stretched elastomeric reservoir (21). This variation in infusion rate is considered clinically acceptable with no hazard to patients (22, 23). Using medications with relatively long half-life and the time pattern of postoperative pain make the use of disposable pumps justified for postoperative analgesia. Postoperative pain generally decreases over time; therefore, a declining rate of infusion may be best suit the analgesic requirement of patients. However, this flow pattern may provide an inadequate analgesic delivery during the following hours of pump administration, and, consequently, unsatisfactory analgesia. Both the patients and clinicians should consider this characteristic flow pattern to maximize patient safety and satisfaction.

### 5.1. Study Limitations

Although the structure of all elastomeric infusion pumps is similar, they are different in type and number of elastomeric layers, hard versus soft shell and the calibration conditions; all of these affect the delivery rate. Therefore, the findings of this study may not be safely generalized to other similar products available. Another limitation of this study was its in vitro nature. Laboratory experiments provide controlled and uniform conditions for all measurements; however, the results may be actually different from in vivo findings. The presence of backpressure in in vivo conditions and using various possible solutions with different viscosities in the container are examples of variables that may influence the results. 

In conclusion, elastomeric pumps can provide accurate delivery rate with consistent performance after repeated filling. This characteristic beside the declining pattern of flow rate makes the disposable devices a promising choice to provide postoperative analgesia. Clinicians should consider the infusion profile of available devices and consider it in selecting a pump for a certain clinical situation. To select an optimal infusion pump, such variables as acceptable flow rate accuracy, delivery-rate profile, desired infusion duration and the volume of reservoir should be taken into account.
